# Surgical Management Strategies for Pericardial Effusion—A Systematic Review

**DOI:** 10.3390/jcm14144985

**Published:** 2025-07-14

**Authors:** Ruman K. Qasba, Busra Cangut, Amnah Alhazmi, Javeria Naseer, Ayesha Mubasher, Sriharsha Talapaneni, Maurish Fatima, Afsheen Nasir, Shanzil Shafqat, Shreya Avilala, Irbaz Hameed

**Affiliations:** 1 Division of Cardiac Surgery, Department of Surgery, Yale University School of Medicine, New Haven, CT 06510, USA; rumankhurshid@gmail.com (R.K.Q.); amnahralhazmi@gmail.com (A.A.); jnmardan@gmail.com (J.N.); st1222@georgetown.edu (S.T.); maurishfatima16@gmail.com (M.F.); afsheen.nasir@yale.edu (A.N.); anzil.shafqat21@dimc.duhs.edu.pk (S.S.); avila082@umn.edu (S.A.); 2 Icahn School of Medicine at Mount Sinai, New York, NY 10029, USA; busra.cangut@mountsinai.org

**Keywords:** pericardial effusion, subxiphoid pericardial window, video assisted thoracoscopic surgery (VATS)

## Abstract

**Objectives**: Pericardial effusion is the accumulation of excess fluid in the pericardial sac. The etiology is multi-factorial and different techniques are used for management, including subxiphoid approaches, anterior and lateral thoracotomies, video-assisted thoracic surgery (VATS), and percutaneous pericardiocentesis. We evaluate the surgical management strategies for pericardial effusion and their outcomes in this systematic review. **Methods**: A systematic literature review was performed to identify studies on the surgical management of pericardial effusion from inception to February 2024 using PubMed, Cochrane, and Scopus. Articles were independently assessed by two reviewers, with discrepancies resolved by the senior author. Articles were considered for inclusion if they described different pericardial effusion surgical management techniques. Baseline patient characteristics and procedural and outcome variables were extracted. **Results**: A total of 27 studies comprising 2773 patients were evaluated. The median age was 56.2 years (interquartile range 47–62.2). The most common etiologies of pericardial effusion were malignancy (31.0%), post-cardiac surgery (18.7%), and idiopathic (15.4%). Other causes included uremia (9.6%), infection (9.6%), and autoimmune disease (4.2%). The subxiphoid pericardial window was the most common approach (82.6%), followed by anterior and lateral thoracotomy (12.0%), and median sternotomy (0.6%). At median follow-up of 24 months, the most frequent post-procedural complications were recurrence of effusion (10.5%), arrhythmias (2.7%), and pneumonia (0.7%). **Conclusions**: Subxiphoid pericardial window is the most common approach for draining pericardial effusions. Prognosis depends on both the underlying etiology and the chosen drainage strategy. Treatment should be tailored to individual patients, considering patient comorbidities and the specific etiology.

## 1. Introduction

Pericardial effusion is the accumulation of fluid in the pericardial sac exceeding a physiologic amount of 10–50 mL [1]. As accumulation progresses, the increased pressure on the cardiac chambers impairs cardiac filling and diminishes stroke volume. In severe cases, cardiac tamponade may develop, especially with the acute accumulation of pericardial effusion [1].

The etiologies of pericardial effusion are multifactorial and the clinical presentation, as well as the rate and volume of fluid accumulation, vary by etiology [1]. In North America and Western Europe, idiopathic pericarditis is the most common etiology resulting in inflammation-related pericardial effusion. In developing countries, tuberculosis remains the predominant cause [2,3]. Other common causes include viral or bacterial infections, malignancies, autoimmune disorders, hypothyroidism, chest trauma, and post-cardiac surgery complications.

Previous studies suggest that the prognosis of pericardial effusion is associated with etiology and drainage strategy [1,4,5]. The 2015 ESC Guidelines for the diagnosis and management of pericardial diseases recommend treatment of pericardial effusion by targeting the underlying disease, as 60% of cases are associated with a known disease [1]. Procedural intervention is recommended if symptoms progress, cardiac tamponade develops, diagnostic sampling is required, or initial management fails [1,6]. Percutaneous pericardiocentesis is preferred in unstable patients with large effusions or complications due to cardiac tamponade [3]. Data on current surgical treatment practices, as well as outcomes, are limited and practices are discordant.

Previous studies have been limited to specific approaches or indications, focusing on selected outcomes. This study provides a comprehensive review of pericardial effusion surgical management in relation to etiology and detailed perioperative outcomes for each technique.

## 2. Materials and Methods

**Figure 1:**
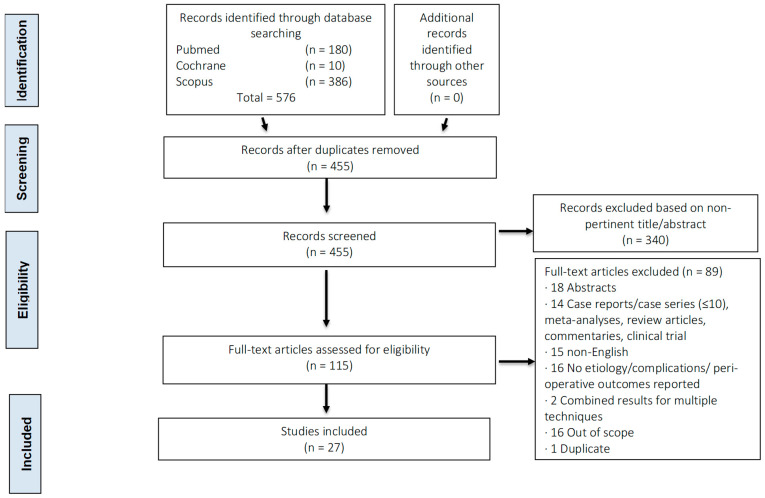
PRISMA Flowchart.

This systematic review was reported following the Preferred Reporting Items for Systematic Reviews and Meta-Analyses (PRISMA) guidelines and registered with PROSPERO (CRD42024527325; www.crd.york.ac.uk/prospero (accessed on 1 April 2024) (Figure 1).

### 2.1. Search Strategy

An electronic search of PubMed, Cochrane, and Scopus was conducted for pericardial effusion surgical management studies published in English from inception to February 2024. We used combinations of the following keywords and medical subject headings (MeSH): “subxiphoid pericardial window” OR “subxiphoid approach” OR “subxiphoid technique” OR “subxiphoid surgery” OR “VATS pericardial window” OR “video assisted thoracoscopic surgery pericardial window” OR “VATS approach” OR “VATS technique” OR “VATS surgery” AND “pericardial effusion” OR “pericardial fluid” OR “pericardial drainage” OR “pericardial tamponade”. Articles were considered for inclusion if they were in English and described different pericardial effusion surgical draining techniques and their outcomes. Our review included patients of all ages, including the pediatric population.

Case reports, case series, literature reviews, abstracts with no access to full articles, and cadaver and animal studies were excluded. For studies that described or compared different techniques, data for individual techniques were separately extracted.

Three authors (J.N., R.K.Q., A.A.) independently reviewed the studies for inclusion. After deduplication, title and abstract screenings were performed independently by the authors. Studies meeting the inclusion criteria were retrieved and screened for the full text. Discrepancies were resolved by the senior author (I.H.).

### 2.2. Data Extraction

Three reviewers independently (J.N., R.K.Q., A.A.) performed data extraction from all the included studies into a pre-piloted data extraction form. Variables included study data (author, country, institution, sample size, study design, study period, and year of publication), patient characteristics (age, gender, and comorbidities, including diabetes, hypertension, coronary artery disease, malignancy, etc.), etiology, procedure details (type of pericardial window, anesthesia, duration of tube drainage, and amount and type of fluid drained), and complications (Table 1 and Table 2, Table S1).

### 2.3. Assessment of Risk of Bias

Two authors (J.N., R.K.Q.) independently performed a quality assessment of the included studies using the Newcastle–Ottawa scale checklist to assess/the quality of nonrandomized studies in meta-analyses. Discrepancies were resolved by the senior author (I.H.) (Table S2).

### 2.4. Pericardial Effusion Surgical Drainage Techniques

#### 2.4.1. The Subxiphoid Pericardial Window

**Figure 2:**
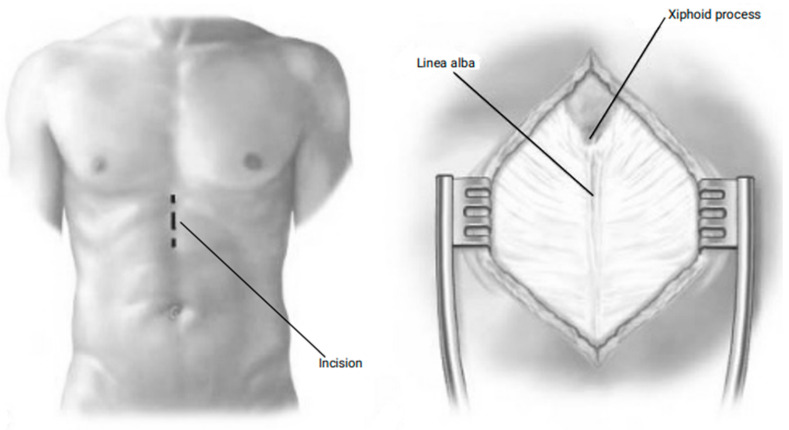
The subxiphoid pericardial window approach.

A vertical midline incision, approximately 4 cm in length, is created over the xiphoid process and upper abdomen. The linea alba is divided, and the xiphoid is either removed or retracted upward, along with the distal sternum. The diaphragm is carefully dissected from the undersurface of the sternum and xiphoid, and any fat covering the pericardium is removed. The pericardium is then opened under direct visualization, and all fluid is aspirated. A drain is placed, set to suction, and the wound is closed (Figure 2).

#### 2.4.2. Anterior and Lateral Thoracotomy

**Figure 3:**
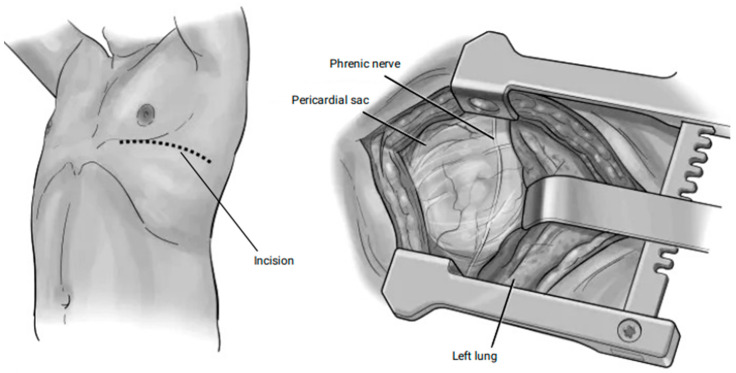
Anterior and lateral thoracotomy approach.

An incision is made to create a simple pericardial window, typically positioned anterior to the phrenic nerve. Following the evacuation of fluid, chest tubes are placed, with one directed posterolaterally and another anteriorly, exiting the pleural space through lower intercostal stab incisions. The procedure concludes with the closure of the incision (Figure 3).

#### 2.4.3. VATS

**Figure 4:**
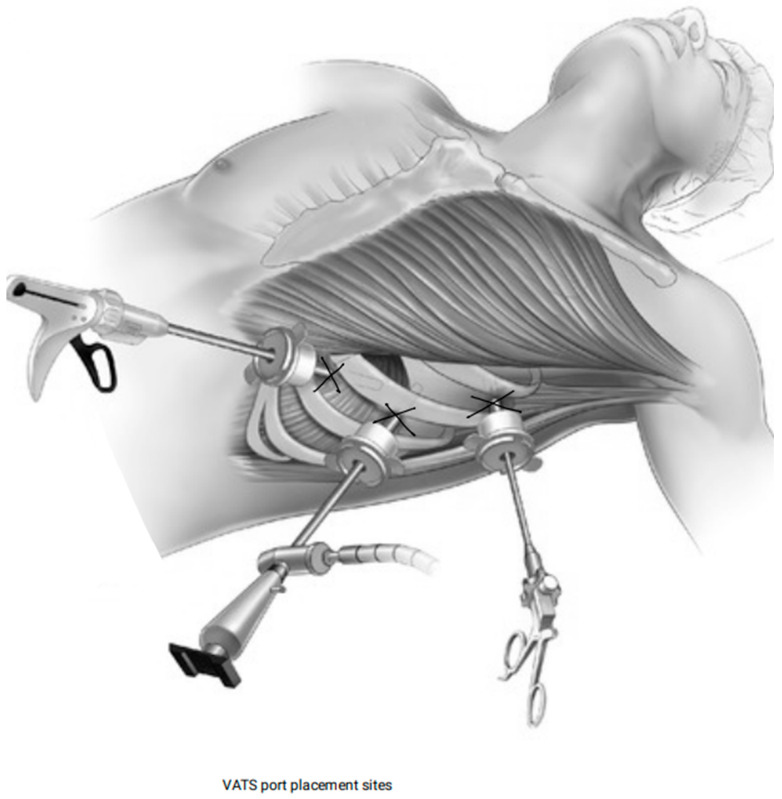
Video-Assisted Thoracoscopic Surgery (VATS) approach.

Video-Assisted Thoracoscopic Surgery (VATS) for pericardial effusion is a minimally invasive procedure to create a pericardial window and drain fluid. Under general anesthesia, the patient is positioned laterally, and 2–3 small incisions are made in the chest wall for the thoracoscope and instruments. After visualizing the pleural cavity, the pericardium is identified, and a small window is created, typically anterior or inferior to the phrenic nerve, to evacuate the effusion. The fluid is collected for diagnostic evaluation, and chest tubes are placed in the pericardial and pleural spaces for continued drainage. The procedure concludes with inspection for hemostasis and closure of the incisions (Figure 4).

#### 2.4.4. Median Sternotomy

**Figure 5:**
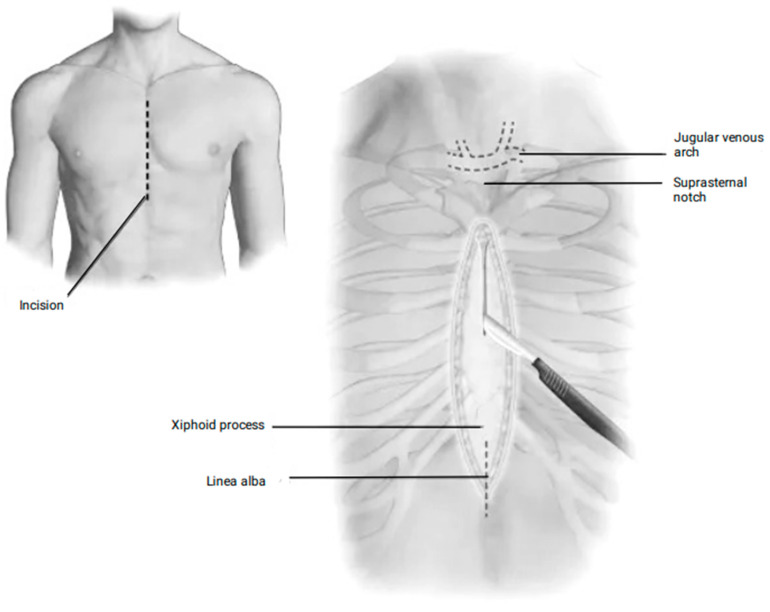
Median sternotomy approach.

A median sternotomy is a surgical procedure in which a vertical incision is made along the midline of the chest to directly open the pericardium and drain the effusion. The procedure concludes with inspection for hemostasis and closure of the incisions (Figure 5).

## 3. Results

### 3.1. Study and Patient Characteristics

A total of 576 records were identified from the initial search strategy, of which 455 were retrieved after deduplication; 340 records were excluded after screening the titles and abstracts, yielding 115 records for the full-text screening. A total of 27 articles comprising 2773 patients met the inclusion and exclusion criteria and were included in the systematic review (Figure 1).

The study population included both pediatric and adult patients, with ages ranging from 1 day to 88 years. The median age was 56.2 years (interquartile range 47–62.2).

### 3.2. Etiology of Pericardial Effusion

**Figure 6:**
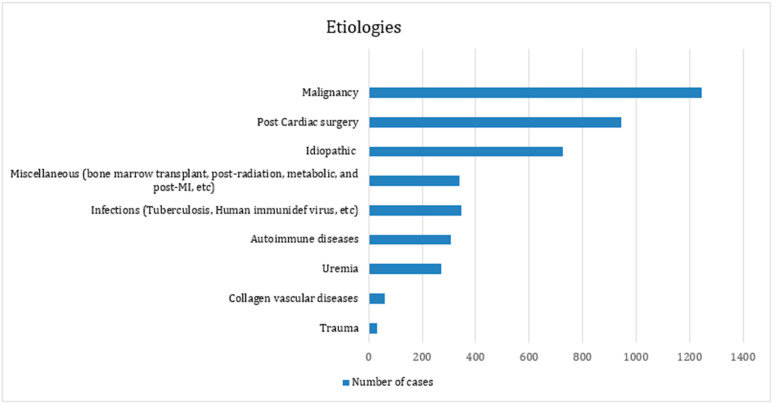
Pericardial effusion etiologies.

The details of the various etiologies of pericardial effusion are reported in Table 1 and Figure 6. The most common etiology was malignancy (31.0%), followed by post-cardiac surgery accumulation (18.7%) and idiopathic etiologies (15.4%). Other etiologies of pericardial effusion included infections (9.6%) such as tuberculosis and HIV, and non-cardiac surgery with causes including autoimmune disease (4.2%) and uremia (9.6%).

### 3.3. Intervention Type

The different techniques for managing pericardial effusion are detailed in Table 1 and Table 2. The subxiphoid pericardial window was the most common approach (82.6%), followed by anterior and lateral thoracotomy (12.0%), VATS (2.9%), and median sternotomy (0.6%).

### 3.4. Outcomes by Type of Intervention

The details of complications encountered with different pericardial effusion management techniques are summarized in Table 1 and Table 2. Of note, patients undergoing anterior or lateral thoracotomy or median sternotomy had no reported intra-operative complications.

### 3.5. Subxiphoid Pericardial Window

#### 3.5.1. Intra-Operative Complications

Among the 2292 patients (82.6%) who underwent drainage via subxiphoid pericardial window, one patient (0.04%) experienced pneumothorax and another (0.04%) developed tension pneumothorax. Additionally, during the creation of the subxiphoid window, there was one episode (0.04%) of intraoperative hypotension and one instance (0.04%) of ventricular fibrillation, which required defibrillation.

#### 3.5.2. Post-Operative (30-Day) Outcomes

A total of 172 patients (7.5%) had recurrent pericardial effusion. Twenty-seven patients (1.17%) had wound infections, fifteen patients (0.6%) had pneumonia, and sixteen patients (0.6%) had renal failure. Additionally, eight patients (0.3%) developed post-procedural arrhythmias, six (0.2%) had a post-operative pneumothorax, five (0.2%) had stroke, and five (0.2%) had post-operative bleeding.

#### 3.5.3. Chest Tube Drainage Amount and Duration of Stay

Patients undergoing subxiphoid pericardial window had chest tubes for 2.0 ± 0.5 to 7.0 ± 6.3 days. The total amount of effusion drainage during this time ranged from 317.0 ± 32.0 to 1131.3 ± 417.0 mL. Patients undergoing the subxiphoid pericardial window approach had an average length of hospital stay of 6.3 ± 1.5 to 13.3 ± 22.9 days.

#### 3.5.4. Mortality

At a mean follow-up of 24 months across the studies, patients receiving subxiphoid pericardial window had a mortality rate of 16.4% (376 deaths).

### 3.6. Anterior or Lateral Thoracotomy Approach

#### 3.6.1. Intra-Operative Complications

While no study in our review specifically reported intra-operative complications associated with this approach, it is important to recognize that this approach is not without risk.

#### 3.6.2. Post-Operative (30-Day Outcomes)

Among the 334 patients (12.0%) who underwent drainage via the anterior or lateral thoracotomy approach, 4 (1.1%) patients undergoing anterior thoracotomy experienced recurrence of pericardial effusion, 2 (0.5%) had post-operative arrhythmias, 1 (0.2%) developed pneumonia, and 3 (0.8%) experienced wound infections.

#### 3.6.3. Chest Tube Drainage Amount and Duration of Stay

Patients undergoing anterior and lateral thoracotomy approach on average drained 452.0 ± 139.0 to 800.0 ± 393.3 ml of effusion, the highest amount compared to the other approaches. The patients had an average length of hospital stay of 9.5 ± 6.0 to 14.9 ± 32.0 days.

#### 3.6.4. Mortality

At a mean follow-up of 24 months across the studies, patients undergoing anterior and lateral thoracotomy approach had a mortality rate of 1.1% (four deaths).

### 3.7. VATS Pericardial Window

#### 3.7.1. Intra-Operative Complications

No study reported any intra-operative complications associated with this approach.

#### 3.7.2. Post-Operative (30-Day Outcomes)

Among the 83 patients (2.9%) who underwent drainage via VATS, 2 (2.4%) had recurrent effusion, 3 (3.6%) developed post-operative arrhythmias, 1 (1.2%) had a pneumothorax, and 1 (1.2%) developed pneumonia. Additionally, one patient (1.2%) experienced a wound infection following VATS.

#### 3.7.3. Chest Tube Drainage Amount and Duration of Stay

Patients undergoing VATS required chest tubes for 1.0 ± 0.5 to 3.4 ± 5.0 days. The total amount of effusion drained during this time ranged from 532.0 ± 106.9 to 735.0 ± 742.0 mL. Patients undergoing VATS had an average length of hospital stay of 1.0 ± 0.5 to 12.4 ± 22.8 days.

#### 3.7.4. Mortality

At a mean follow-up of 24 months across the studies, patients undergoing VATS drainage had a mortality of 14.4% (12 deaths).

## 4. Discussion

### 4.1. Etiology and Outcomes

The etiologies of pericardial effusion vary, and treatment should target the underlying cause. While studies have evaluated specific techniques, a comprehensive comparison between different management approaches and their outcomes remains challenging due to the heterogenous nature of patients with pericardial effusion. In clinical practice, the choice of management often depends on the underlying cause of the effusion, the clinical scenario, and the expertise available locally. Our systematic review of 27 studies (2773 patients) assessing the various etiologies of pericardial effusion in the context of the different surgical approaches and their associated outcomes is the most comprehensive to date. Among the patients included in the study, pericardial effusion of malignant origin and post-cardiac surgery were the most common etiologies, with the subxiphoid pericardial window approach being the most common treatment modality. Recurrence of pericardial effusion was observed in 6.4% of cases (n = 177). The subxiphoid pericardial window approach was associated with the highest rate of effusion recurrence (7.5%), followed by VATS (2.4%), and 1.1% for the left anterior thoracotomy approach.

Also, patients receiving subxiphoid pericardial window had the highest mortality rate, at 16.4%. It must be noted, however, that it is quite likely that the underlying etiology of the pericardial effusion, rather than the drainage strategy itself, contributed to the observed mortality. This distinction is a crucial consideration when interpreting these outcomes.

The etiology of pericardial effusion is associated with several factors. The current narrative literature explores the role of management techniques for pericardial effusions with various underlying etiologies, although specific outcomes are under-reported [5,32]. Consistent with our findings, Sagristà-Sauleda et al. [32] found neoplastic etiology to be the most common cause of pericardial effusion. Similarly, a study by W. Ma et al. [33] identified neoplastic and tuberculosis-related pericardial effusions as the most prevalent causes. Additionally, one of the largest registries of pericardiocentesis, published nearly 20 years ago by the Mayo Clinic, examined the etiologies of pericardial effusion over the last three decades of the twentieth century in a single center [34,35,36]. This study demonstrated a significant shift in the causes of pericardial effusion over time, with post-cardiac surgery, neoplastic causes, and cardiac perforations following invasive procedures being the most frequent during the 1990s [37].

### 4.2. Procedure Selection Based on Etiology

Pericardial drainage procedures can be performed for both diagnostic and therapeutic purposes, particularly in patients with cardiac tamponade. However, in patients without hemodynamic compromise, the diagnostic yield from pericardial fluid or tissue is minimal [38]. A study by Merce et al. [39], which included 71 patients with large pericardial effusion without clinical tamponade, found that pericardial drainage procedures performed in 26 patients had a diagnostic yield of only 7.0%. Additionally, none of the patients developed cardiac tamponade or died as a result of pericardial disease, and no new diagnoses emerged in the 45 patients who did not initially undergo pericardial drainage. Furthermore, moderate or large effusions persisted in only 2 of the 45 patients managed conservatively [39]. Therefore, routine pericardial drainage procedures are not justified in patients without hemodynamic compromise, with some notable exceptions. In our practice, drainage of a pericardial effusion is offered to symptomatic patients or those with hemodynamic compromise. The drainage tubes are typically removed when the volume of drainage is less than 25 mL within 24 h.

The choice of procedure for treating pericardial effusion largely depends on its underlying etiology. For patients with acute idiopathic or viral pericarditis, simple pericardiocentesis is typically sufficient. In cases of purulent or tuberculous pericarditis, surgical drainage through a subxiphoid pericardiotomy is recommended. Neoplastic pericardial effusion poses a greater challenge due to the high risk of fluid reaccumulation. Examining pericardial fluid is necessary to first determine whether the effusion is secondary to neoplastic involvement, or a non-malignant phenomenon related to cancer management, such as previous thoracic irradiation. The primary goals of treatment are to relieve symptoms and prevent fluid reaccumulation, which is common in these patients. Generally, less invasive procedures are preferred, especially for those with advanced disease and poor overall condition. Simple pericardiocentesis alleviates symptoms in most cases, but recurrence of pericardial effusion occurs in as many as 40–50% of patients [40]. Therefore, in cases where recurrence is not a concern, percutaneous pericardiocentesis would be the procedure of choice [32]. Otherwise, therapeutic options for patients with neoplastic pericardial effusion include extended indwelling pericardial catheters, percutaneous pericardiostomy, and the intrapericardial instillation of antineoplastic or sclerosing agents. Additionally, colchicine is frequently used as a medical therapy for pericarditis and small effusions, demonstrating efficacy in reducing inflammation and recurrence [41].

In the studies included in our review, the subxiphoid pericardial window approach was the most commonly utilized surgical method.

### 4.3. Management of Recurrent Effusions and Preventative Strategies

In our review, recurrence of effusion was noted in 10.2% of patients, with differences observed in rate of recurrence across the different drainage techniques. The highest recurrence rate was associated with subxiphoid pericardial window and the lowest rate was associated with the left anterior thoracotomy approach. This may be due to greater control and the control of window size in patients undergoing left anterior thoracotomy and the ability to position the catheter in the effusion pocket under direct vision. However, it is important to note that, despite these recurrence percentages, we do not have exact data on the number of patients who required repeat drainage procedures or the degree of recurrent effusion, as this information was not consistently reported in the included studies.

In a study by Girardi et al. [42], regardless of the method chosen, 3% to 10% of all patients required further intervention for the management of recurrent pericardial effusion. Two major interventions used to prevent the recurrence of malignant pericardial effusions were the use of intrapericardial sclerosing agents, such as thiotepa, and the placement of an intrapericardial catheter for prolonged drainage [43]. Girardi et al. [42] reported that the addition of intrapericardial sclerotherapy appeared to reduce the recurrence rate to an acceptable level. Another study by Maher et al. [44] found that in patients with known malignancy or positive cytologic examination of pericardial fluid, sclerosis appeared to be superior to subxiphoid pericardial window formation and VATS in preventing recurrence.

Indwelling pericardial catheters have a success rate of approximately 75%, defined as the alleviation of tamponade and no need for further procedures. The catheter should be maintained as long as the drainage amount remains greater than 25 mL/day [32]. In a study by Moores et al. [9], the median duration of catheter drainage in their patients was only 2.7 days, significantly less than that reported for open drainage. Another study by Rafique et al. [45] reported that patients with extended catheter drainage had a reduced recurrence rate of 12%, compared to 52% in patients without extended drainage (*p* < 0.001). One possible mechanism by which extended pericardial catheter drainage prevents recurrent effusion and tamponade is through the complete evacuation of fluid and irritation of the pericardium, which enhances apposition of the visceral and parietal pericardium, thereby preventing further recurrence [46,47,48]. Additionally, a study by Sushil et al. [49] recommended that extended pericardial catheter drainage should continue until net pericardial drainage decreases to less than 50 mL/24 h. This practice is advised for all patients to minimize recurrence risk, with an anticipated mean time to catheter removal of approximately 4 days. Tsang et al. [37] also reported the routine practice of leaving the catheter in the pericardial space until there is less than 25–30 mL of drainage during the preceding 24 h period to ensure complete evacuation of the pericardial effusion.

### 4.4. Complications, Length of Hospital Stay, and Mortality Variations

Pericardiocentesis has been associated with various complications, including vasovagal reactions, transient arrhythmias, sinus node dysfunction, and temporary or persistent elevation of the ST or PR segment [50,51,52].

The length of hospital stay for patients undergoing different surgical approaches for pericardial effusion varies significantly [7]. Patients undergoing a subxiphoid pericardial window had a length of hospital stay ranging from 6.3 ± 1.5 to 13.3 ± 22.9 days, as reported in the literature. In contrast, those undergoing anterior and lateral thoracotomy approaches had an average length of hospital stay of 9.5 ± 6.0 to 14.9 ± 32.0 days. VATS demonstrated a broader range of hospital stay durations, from 1.0 ± 0.5 to 12.4 ± 22.8 days. These variations likely reflect differences in procedural invasiveness and postoperative recovery protocols. For example, VATS is typically associated with shorter hospital stays, as its minimally invasive approach helps reduce postoperative pain and promotes quicker recovery [8].

Differences in mortality rates were observed among the procedures, with higher rates associated with subxiphoid pericardial window procedure (16.4%) compared to VATS (14.4%) and anterior and lateral thoracotomy (1.1%). This aligns with the findings reported by O’Brien et al., which indicate that the mortality rate was 13% for patients undergoing the subxiphoid pericardial window technique, compared to zero for those who had the VATS procedure. The prognosis of chronic pericardial effusions is largely determined by the underlying cause. Recent evidence suggests that in patients with idiopathic, chronic (>3 months), large (>2 cm), and asymptomatic pericardial effusions, the outlook is generally favorable. In such cases, a watchful waiting approach appears to be more reasonable and cost-effective compared to routine drainage, which was previously recommended [53]. Malignant pericardial effusions tend to be severe, with most patients presenting with cardiac tamponade. Treatment options remain limited, and the condition is associated with a high mortality rate [54].

The reported mortality rates might reflect the underlying etiology and overall clinical condition of the patients, rather than the procedural approach itself, highlighting the importance of interpreting these findings within the appropriate clinical context. Additionally, while we stated that no intraoperative complications were reported for certain procedures, it is important to acknowledge that the absence of reported complications does not necessarily indicate their nonoccurrence. This lack of reporting in the studies included may reflect incomplete documentation.

### 4.5. Limitations

Some limitations of our study need to be highlighted. Most of the studies were retrospective, and certain variables, such as effusion volume, serological tests, or clinical characteristics, were not systematically recorded and thus could not always be obtained. We acknowledge that the retrospective nature of the included studies and their reliance on descriptive statistics limit causal inference, as confounding factors such as underlying etiology and patient selection cannot be fully adjusted for.

## 5. Conclusions

The prognosis of pericardial effusion is associated with its underlying etiology, which should guide the choice of management approach. The first-line treatment should target the underlying etiology of the effusion. In cases where conservative management fails, percutaneous pericardiocentesis remains a viable option, particularly for patients with advanced disease. Given the high recurrence rate of effusions following percutaneous pericardiocentesis, other approaches, including the subxiphoid pericardial window, thoracotomy, or VATS, warrant consideration especially if a concomitant biopsy is required or risk of effusion recurrence is high. The choice of intervention should ultimately be individualized, taking into account the patient’s comorbidities and previous history of pericardial effusion, as well as the size and location of the effusion.

## Figures and Tables

**Table 1:** Details of etiology of pericardial effusion in the included studies.

			Etiology of Pericardial Effusion				
Study	Technique	Total Procedures (N)	Uremia (N)	Trauma (N)	Malignancy (N)	Collagen Vascular Disease (N)	Tuberculosis (N)
**Studies Investigating Individual Pericardial Effusion Management Techniques**							
**Palatianos, 1989 [7]**	Subxiphoid Pericardial window	41	9	0	14	0	2
**Trigt, 1993 [8]**	Subxiphoid Pericardial window	57	7	0	13	7	0
**Moores, 1995 [9]**	Subxiphoid Pericardial window	155	0	0	82	10	3
**Sarigül, 1999 [10]**	Subxiphoid Pericardial window	305	93	0	41	0	21
**Becit, 2003 [11]**	Subxiphoid Pericardial window	240	101	9	34	0	25
**Daugirdas, 1986 [12]**	Subxiphoid Pericardial window	16	0	0	0	0	0
**Dosios, 2003 [13]**	Subxiphoid Pericardial window	104	0	0	46	3	3
**Porte, 1999 [14]**	Subxiphoid Pericardial window	114	0	0	43	0	0
**Wall, 1992 [15]**	Subxiphoid Pericardial window	57	7	0	13	7	3
**Olsen, 1991 [16]**	Left anterior thoracotomy	60	4	1	28	0	1
**Celik, 2012 [17]**	Left anterior thoracotomy	48	0	0	26	0	2
**Çardak, 2023 [18]**	VATS	20	NR	NR	NR	NR	NR
**Georghiou, 2005 [19]**	VATS	18	0	0	3	0	0
**Altman, 2015 [20]**	Anterior parasternal approach	30	0	0	9	0	0
**Olson, 1995 [21]**	Pericardial-peritoneal window through a subxiphoid approach	33	3	0	18	3	0
**Salim, 2018 [22]**	Total	30	0	0	0	0	0
	Subxiphoid Pericardial window	15	0	0	0	0	0
	VATS	15	0	0	0	0	0
**Piehler, 1985 [23]**	Total	145	2	1	72	9	0
	Subxiphoid Pericardial window	13	NR	NR	NR	NR	NR
	Left anterior thoracotomy	118	NR	NR	NR	NR	NR
	Median sternotomy	7	NR	NR	NR	NR	NR
	Right thoracotomy	7	NR	NR	NR	NR	NR
**Park, 1991 [24]**	Total	28	0	0	19	0	0
	Subxiphoid Pericardial window	10	0	0	5	0	0
	Left anterior thoracotomy	6	0	0	14	0	0
	Median sternotomy	12					
**Wilkes,1995 [25]**	Total	127	1	0	70	0	0
	Subxiphoid Pericardial window	85	NR	NR	NR	NR	NR
	Thoracotomy with pleuro-pericardial window	7	NR	NR	NR	NR	NR
	Anterior thoracotomy with pericardiotomy	6	NR	NR	NR	NR	NR
	Laparotomy with pericardiotomy	2	NR	NR	NR	NR	NR
**Naunheim, 1991 [4]**	Total	131	24	7	38	8	0
	Subxiphoid Pericardial window	53	4	NR	16	4	0
	Transthoracic	78	20	NR	22	4	0
**Allen, 1999 [26]**	Total	117	9	NR	75	4	0
	Subxiphoid Pericardial window	94	NR	NR	NR	NR	NR
**O’Brien, 2005 [27]**	Total	71	0	0	50	3	0
	Subxiphoid Pericardial window	56	NR	NR	NR	NR	NR
	VATS	15	NR	NR	NR	NR	NR
**Langdon, 2016 [4]**	Total	179	NR	NR	NR	NR	NR
	Subxiphoid Pericardial window	127	NR	NR	NR	NR	NR
	Left anterior thoracotomy	52	NR	NR	NR	NR	NR
**Balla, 2020 [28]**	Total	46	8	0	22	4	0
	Subxiphoid Pericardial window	31	NR	NR	NR	NR	NR
	Trans pleural	15	NR	NR	NR	NR	NR
**McDonald, 2003 [29]**	Total	246	0	11	79	0	0
	Subxiphoid Pericardial window	150	0	8	52	0	0
**Horr, 2017 [30]**	Total	1281	0	0	215	0	0
	Subxiphoid Pericardial window	521	0	0	75	0	0
**Muhammad, 2011 [31]**	Total	30	6	0	17	0	0
	Subxiphoid	15	4	0	7	0	0
	VATS	15	2	0	10	0	0
		**Etiology of Pericardial Effusion**					
**Study**	**Post Cardiotomy/Cardiac Catheterization** **(N)**	**Idiopathic** **(N)**	**Infection** **(N)**	**Autoimmune Disease** **(N)**		**Radiation/Irradiation** **(N)**	**Other** **(N)**
**Studies Investigating Individual Pericardial Effusion Management Techniques**							
**Palatianos, 1989 [7]**	0	9	0	0		0	7
**Trigt, 1993 [8]**	0	4	15	0		8	5
**Moores, 1995 [9]**	17	14	17	0		0	12
**Sarigül, 1999 [10]**	0	29	54	0		0	67
**Becit, 2003 [11]**	0	52	12	0		0	7
**Daugirdas, 1986 [12]**	0	0	0	0		0	16
**Dosios, 2003 [13]**	2	27	9	0		1	13
**Porte, 1999 [14]**	0	33	10	0		20	8
**Wall, 1992 [15]**	0	4	8	0		8	8
**Olsen, 1991 [16]**	0	13	2	3		0	8
**Celik, 2012 [17]**	0	15	2	0		2	1
**Çardak, 2023 [18]**	NR	NR	NR	NR		NR	NR
**Georghiou, 2005 [19]**	0	11	0	0		0	4
**Altman, 2015 [20]**	0	17	0	0		0	4
**Olson,1995 [21]**	0	9	0	0		0	0
**Studies investigating multiple pericardial effusion management techniques**							
**Salim, 2018 [22]**	0	0	0	0		0	30
	0	0	0	0		0	15
	0	0	0	0		0	15
**Piehler, 1985 [23]**	0	42	4	0		15	0
	NR	NR	NR	NR		NR	NR
	NR	NR	NR	NR		NR	NR
	NR	NR	NR	NR		NR	NR
	NR	NR	NR	NR		NR	NR
**Park, 1991 [24]**	0	5	0	0		4	0
	0	2	0	0		3	0
	0	3	0	0		1	0
**Wilkes, 1995 [25]**	0	42	6	0		4	4
	NR	NR	NR	NR		NR	NR
	NR	NR	NR	NR		NR	NR
	NR	NR	NR	NR		NR	NR
	NR	NR	NR	NR		NR	NR
**Naunheim, 1991 [4]**	0	15	27	0		9	3
	0	NR	16	0		3	15
	0	NR	22	0		6	10
**Allen, 1999 [26]**	4	19	3	0		0	3
	NR	NR	NR	NR		NR	NR
**O’Brien, 2005 [27]**	2	12	0	0		0	4
	NR	NR	NR	NR		NR	NR
	NR	NR	NR	NR		NR	NR
**Langdon, 2016 [4]**	NR	NR	NR	NR		NR	NR
	NR	NR	NR	NR		NR	NR
	NR	NR	NR	NR		NR	NR
**Balla, 2020 [28]**	0	12	0	0		0	0
	NR	NR	NR	NR		NR	NR
	NR	NR	NR	NR		NR	NR
**McDonald, 2003 [29]**	43	0	27	31		0	55
	27	0	19	14		0	30
**Horr, 2017 [30]**	656	190	70	265		0	187
	336	50	20	100		0	17
**Muhammad, 2011 [31]**	0	7	0	0		0	0
	0	4	0	0		0	0
	0	3	0	0		0	0

NR, Not reported; VATS, video-assisted thoracoscopic surgery. Footnote: Park, 1991, reported the combined etiologies of left anterior thoracotomy and median sternotomy as group 2.

**Table 2:** Perioperative details.

				Intraoperative Details								
Study	Technique	Operative Pericardial Effusion Drainage (mL)		Post Operative Pericardial Effusion Drainage. (mL)		Operative Time (min)		Chest Tube Drainage (Day)		Intraoperative Complications		
	**Studies Investigating Individual Pericardial Effusion Management Techniques.**											
**Palatianos, 1989 [7]**	Subxiphoid pericardial window	NR		NR		NR		NR		NR		
**Trigt, 1993 [8]**	Subxiphoid pericardial window	NR		NR		NR		NR		NR		
**Moores, 1995 [9]**	Subxiphoid pericardial window	NR		NR		NR		NR		NR		
**Sarigül, 1999 [10]**	Subxiphoid pericardial window	Benign effusion {975.3 ± 48.5}, Malignant effusion {1131.3 ± 97.5}		NR		NR		NR		NR		
**Becit, 2003 [11]**	Subxiphoid pericardial window	696.0 ± 32.0		NR		NR		NR		NR		
**Daugirdas, 1986 [12]**	Subxiphoid pericardial window	Median 600 (300–1500)		NR		NR		(2.0–4.0)		NR		
**Dosios, 2003 [13]**	Subxiphoid pericardial window	NR		NR		(35.0–50.0)		NR		NR		
**Porte, 1999 [14]**	Subxiphoid pericardial window	750 mL (range 50 mL in a patient with previous transcutaneous drainage, to 1600 mL).		NR		36 min (range: 21 ± 74)		5 days (range 4 ± 6		PCS was complete in 112 of the 114 patients (98%). The two incomplete explorations were due to a cardiac arrest during the induction of anesthesia in one case, and the presence of neoplastic tissue hindering introduction of the pericardioscope in the other.		
**Wall, 1992 [15]**	Subxiphoid pericardial window	NR		NR		NR		NR		NR		
**Olsen, 1991 [16]**	Left anterior thoracotomy	800.0 {250.0–2100.0}		NR		NR		NR		NR		
**Celik, 2012 [17]**	Left anterior thoracotomy	862.50 ± 390.37		NR		28.3 ± 3.8		6.0 ± 1.5		NR		
**Çardak, 2023 [18]**	VATS	700.0 ± 307.0		NR		44.0 ± 13.0		1.0 {1.0–1.0}		NR		
**Georghiou, 2005 [19]**	VATS	NR		NR		Mean 46 (30–160)		Mean 2.3 (1.0–5.0)		0.0		
**Altman, 2015 [20]**	Anterior parasternal approach	700.0 ± 139.0		NR		73.0 ± 21.0		NR		NR		
**Olson, 1995 [21]**	Pericardial–peritoneal window through a subxiphoid approach	500.0 {50.0–1600.0}		NR		78.0 {25.0–143.0}		NR		(1 hypotension without sequela) (3—chest tubes for later pleurodesis coexisting malignant pleural effusions) intraoperative entry into left pleural space (n = 1)		
	Studies investigating multiple pericardial effusion management techniques.											
**Salim, 2018 [22]**	Subxiphoid pericardial window	591.8 ± 154.4		NR		34.5 ± 2.7		3.34 d- + 0.5		NR		
	VATS	532.1 ± 106.9		NR		58.9 ± 4.6		2.4 ± 0.5		NR		
**Piehler, 1985 [23]**	Total	NR		NR		NR		NR		NR		
	Subxiphoid pericardial window	NR		NR		NR		NR		NR		
	Left anterior thoracotomy	NR		NR		NR		NR		NR		
	Median sternotomy	NR		NR		NR		NR		NR		
	Right thoracotomy	NR		NR		NR		NR		NR		
**Park, 1991 [24]**	Subxiphoid pericardial window	NR		NR		NR		3.2 (1.0–5.0)		NR		
	Transthoracic (left anterior thoracotomy median sternotomy)	NR		NR		NR		7.3 (3.0–16.0)		NR		
**Wilkes, 1995 [25]**	Total	509.0 (5.0–2300.0)		NR		NR		NR		NR		
	Subxiphoid pericardial window	NR		NR		NR		NR		NR		
	Thoracotomy with pleuro-pericardial window	NR		NR		NR		NR		NR		
	Anterior thoracotomy with pericardiotomy	NR		NR		NR		NR		NR		
	Laparotomy with pericardiotomy	NR		NR		NR		NR		NR		
**Naunheim, 1991 [4]**	Subxiphoid pericardial window	455.0 ± 388.0		NR		NR		5.4 ± 6.3		NR		
	Transthoracic (sternotomy and anterior thoracotomy)	487.0 ± 359.0		NR		NR		4.0 ± 2.5		NR		
**Allen, 1999 [26]**	Subxiphoid pericardial window	NR		NR		NR		5.0		NR		
**O’Brien, 2005 [27]**	Subxiphoid pericardial window	433.0 ± 417.0		NR		81.1 ± 25.5		4.0 ± 1.6		The pleural chest tube was placed intraoperatively for a clinical tension pneumothorax after pericardial drainage		
	VATS	735.0 ± 742.0		NR		117.1 ± 32.4		3.3 ± 1.4		Two required additional chest tubes for pneumothorax after chest tube removal, 1 was discharged home with a Heimlich valve for ongoing air leak from injury to a trapped lung, and 1 was readmitted for drainage from a chest tube site that was self-limited		
**Langdon, 2016 [4]**	Subxiphoid pericardial window	512.0 ± 303.0		NR		NR		NR		NR		
	Left anterior thoracotomy	452.0 ± 267.0		NR		NR		NR		NR		
**Balla, 2020 [28]**	Total	NR		NR		NR		NR		NR		
	Subxiphoid pericardial window	500.0 {413.0–600.0}		NR		165.0 {96.0–218.0}		7.0 {6.0–9.0}		NR		
	Trans pleural	450.0 {400.0–575.0}		NR		96.0 {95.0–208.0}		4.0 {4.0–6.0}		NR		
**McDonald, 2003 [29]**	Subxiphoid pericardial window	317.0 ± 132.0		NR		NR		4.5 ± 2.7		Single episode of ventricular fibrillation requiring defibrillation		
**Horr, 2017 [30]**	Surgical pericardial window	NR		NR		NR		2.9 ± 2.2		NR		
**Muhammad, 2011 [31]**	Subxiphoid pericardial window	NR		NR		75.2 ± 25.4		4.1 ± 1.4		NR		
	VATS	NR		NR		111.3 ± 30.7		3.4 ± 1.5		NR		
		Postoperative Outcomes										
**Study**	**Technique**	**Length of** **Hospital Stay (Day)**	**30-Day** **Early Mortality** **(N)**	**30 Days < Late Mortality** **(N)**	**Pericardial Effusion Recurrence** **(N)**	**Pneumonia** **(N)**	**Cardiac Arrhythmia** **(N)**	**Renal Failure** **(N)**	**Wound Infection** **(N)**	**Bleeding** **(N)**	**Stroke** **(N)**	**Other** **(N)**
	**Studies Investigating Individual Pericardial Effusion Management Techniques**											
**Palatianos, 1989 [7]**	Subxiphoid pericardial window	NR	8.0	7.0	1.0	NR	2.0	NR	NR	NR	NR	NR
**Trigt, 1993 [8]**	Subxiphoid pericardial window	NR	7.0	21.0	9.0	NR	NR	NR	NR	NR	NR	2.0
**Moores, 1995 [9]**	Subxiphoid pericardial window	NR	31.0	NR	4.0	NR	NR	NR	NR	NR	NR	NR
**Sarigül, 1999 [10]**	Subxiphoid pericardial window	NR	50.0	NR	31.0	11.0	NR	13.0	3.0	NR	NR	34.0
**Becit, 2003 [11]**	Subxiphoid pericardial window	6.3	3.0	14.0	24.0	NR	NR	NR	12.0	NR	NR	NR
**Daugirdas, 1986 [12]**	Subxiphoid pericardial window	NR	NR	12.0	1.0	NR	NR	NR	3.0	NR	NR	5.0
**Dosios, 2003 [13]**	Subxiphoid pericardial window	NR	17.0	NR	2.0	NR	9.0	NR	NR	NR	NR	5.0
**Porte, 1999 [14]**	Subxiphoid pericardial window	5 days (range: 4 ± 9)	4.0	81.0	5.0	2.0	36.0	NR	5.0	NR	NR	NR
**Wall, 1992 [15]**	Subxiphoid pericardial window	NR	7.0	16.0	7.0	NR	NR	NR	NR	NR	NR	2.0
**Olsen, 1991 [16]**	Left anterior thoracotomy	NR	10.0	NR	NR	NR	NR	NR	NR	NR	NR	NR
**Celik, 2012 [17]**	Left anterior thoracotomy	9.5 ± 7.2	4.0	NR	1.0	1.0	1.0	NR	2.0	NR	NR	3.0
**Çardak, 2023 [18]**	VATS	1.0 {1.0–2.0}	2.0	NR	NR	NR	NR	NR	NR	NR	NR	NR
**Georghiou, 2005 [19]**	VATS	Mean 6.4 (3–16)	1.0	NR	NR	NR	1.0	NR	NR	NR	NR	NR
**Altman, 2015 [20]**	Anterior parasternal approach	10.0 ± 6.8	8.0		NR	4.0	3.0	6.0	NR	NR	NR	21.0
**Olson,1995 [21]**	Pericardial–peritoneal window through a subxiphoid approach	9.0 {3.0–47.0}	3.0	1.0	1.0	2.0	2.0	NR	NR	NR	NR	3.0
	Studies investigating multiple pericardial effusion management techniques.											
**Salim, 2018 [22]**	Subxiphoid pericardial window	13.3 ± 1.1	NR	NR	5.0	1.0	4.0	NR	2.0	NR	NR	NR
	VATS	8.7 ± 0.5	NR	NR	1.0	1.0	3.0	NR	1.0	NR	NR	1.0
**Piehler, 1985 [23]**	Total	NR	18.0	NR	NR	NR	NR	NR	NR	NR	NR	NR
	Subxiphoid pericardial window	NR	NR	NR	NR	NR	NR	NR	NR	NR	NR	NR
	Left anterior thoracotomy	NR	NR	NR	NR	NR	NR	NR	NR	NR	NR	NR
	Median sternotomy	NR	NR	NR	NR	NR	NR	NR	NR	NR	NR	NR
	Right thoracotomy	NR	NR	NR	NR	NR	NR	NR	NR	NR	NR	NR
**Park, 1991 [24]**	Subxiphoid pericardial window	10.3 (4.0–29.0)	NR	10.0	NR	NR	NR	NR	NR	NR	NR	NR
	Transthoracic (left anterior thoracotomy, median sternotomy)	14.9 (6.0–32.0)	NR	16	NR	1.0	1.0	1.0	NR	NR	NR	15.0
**Wilkes,1995 [25]**	Total	NR	NR	NR	NR	NR	NR	NR	NR	NR	NR	NR
	Subxiphoid pericardial window	NR	1%	NR	NR	NR	NR	NR	NR	NR	NR	5% overall
	Thoracotomy with pleuro-pericardial window	NR	NR	NR	NR	NR	NR	NR	NR	NR	NR	NR
	Anterior thoracotomy with pericardiotomy	NR	NR	NR	NR	NR	NR	NR	NR	NR	NR	NR
	Laparotomy with pericardiotomy	NR	NR	NR	NR	NR	NR	NR	NR	NR	NR	NR
**Naunheim, 1991 [4]**	Subxiphoid pericardial window	11.5 ± 11.2	8.0	NR	3.0	3.0	NR	3.0	2.0	NR	NR	42.0
	Transthoracic (sternotomy and anterior thoracotomy)	14.4 ± 12.7	10.0	NR	3.0	7.0	NR	4.0	1.0	1.0	1.0	78.0
**Allen, 1999 [26]**	Subxiphoid pericardial window	NR	0.0	NR	1.0	NR	NR	NR	NR	1.0	NR	NR
**O’Brien, 2005 [27]**	Subxiphoid pericardial window	10.4 ± 12.2	7.0	43.0	5.0	NR	NR	NR	NR	NR	NR	29.0
	VATS	12.4 ± 22.8	0.0	9.0	1.0	NR	NR	NR	NR	NR	NR	7.0
**Langdon, 2016 [4]**	Subxiphoid pericardial window	11.0 ± 7.5	9.0	NR	NR	NR	NR	NR	NR	NR	NR	NR
	Left anterior thoracotomy	11.1 ± 9.5	4.0	NR	NR	NR	NR	NR	NR	NR	NR	NR
**Balla, 2020 [28]**	Total	NR	9.0	NR	8.0	NR	3.0	NR	NR	NR	NR	7.0
	Subxiphoid pericardial window	9.0 {7.0–11.0}	5.0	NR	NR	NR	NR	NR	NR	NR	NR	NR
	Trans pleural	7.0 {6.0–12.0}	3.0	NR	NR	NR	NR	NR	NR	NR	NR	NR
**McDonald, 2003 [29]**	Subxiphoid pericardial window	NR	16.0	NR	7.0	NR	NR	NR	NR	NR	NR	NR
**Horr, 2017 [30]**	Surgical pericardial window	NR	23.0	NR	52.0	NR	NR	NR	NR	5.0	5.0	28.0
**Muhammad, 2011 [31]**	Subxiphoid pericardial window	12.3 ± 22.6	NR	NR	NR	NR	NR	NR	NR	NR	NR	1.0
	VATS	10.2 ± 12.1	NR	NR	NR	NR	NR	NR	NR	NR	NR	NR

NR, not reported; median {range}; {median ± standard deviation; mean (range); mean ± standard deviation. Footnote: Altman, 2015, only reported mortality for the first three months post operation. Footnote: Wilkes, 1995, reported outcomes in percentages, without including raw numbers.

## Data Availability

Data is available upon request from authors.

## Supplementary Materials

The following supporting information can be downloaded at: https://www.mdpi.com/article/10.3390/jcm14144985/s1. Table S1: Summary of included studies; Table S2: Newcastle ottawa scale for observational studies.

## Abbreviations

The following abbreviations are used in this manuscript:

VATS	Video-Assisted Thoracoscopic Surgery (VATS)
HIV	Human Immunodeficiency Virus
PRISMA	Preferred Reporting Items for Systematic Reviews and Meta-Analyses
ESC	European Society of Cardiology
