# Supplementary material for: Surgical Management Strategies for Pericardial Effusion—A Systematic Review
Source: J Clin Med. 2025 Jul 14;14(14):4985. doi: 10.3390/jcm14144985 (PMC12295354; doi:10.3390/jcm14144985)
Supplement: Supplementary file 1 [file jcm-14-04985-s001.zip › jcm-3734073-supplementary.pdf]

Supplementary Table S1: Summary of included studies

Study	Study design	Country	Institution	Study period	Patients (N)
Studies investigating individual pericardial effusion management techniques.					
Palatianos, 1989	Retrospective	United States	University of Miami school of Medicine	1972 - 1986	41
Olsen, 1991	Retrospective	Denmark	Gentofte Hospital	1976 - 1990	60
Trigt, 1993	Prospective	United States	Duke University Medical Center	20 months (specific dates are not reported)	57
Becit, 2003	Retrospective	Turkey	Atatürk University School of Medicine	1990-2000	240
Moores, 1995	Retrospective	United States	Albany Medical Center, Rush-Presbyterian St. Luke's Medical Center	1988 - 1993	155
Olson,1995	Retrospective	United States	Mary Imogene Bassett Hospital	1983-1993	33
Sarigül, 1999	Retrospective	Turkey	Faculty of Medicine, Hacettepe University	1984-19996	305
Celik, 2012	Retrospective	Turkey	Siyami Ersek Thoracic and Cardiovascular Surgery Training and Research Hospital	2002-2008	48
Altman, 2015	Retrospective	Israel	Galilee Medical Center	2001-2011	30
Sigusch, 2022.	Retrospective	Germany	Heinrich-Braun-Klinikum	2008-2021	26
Çardak, 2023	Retrospective	Turkey	Kartal Kosuyolu Education and Research Hospital,	2021-2022	20
Studies investigating multiple pericardial effusion management techniques					
Salim, 2018	Prospective	Egypt	Faculty of Medicine Benha University	2015-2017	
Piehler, 1985	Retrospective	United States	Mayo Clinic and Mayo Foundation	1960 - 1983	145
Park, 1991	Retrospective	United States	Loma Linda University Medical Center	1978-1988	28
Wilkes,1995	Retrospective	United States	Roswell Park Comprehensive Cancer Center	1968 - 1994	127
Naunheim, 1991	Retrospective	United States	St. Louis University Medical Center and Indiana University School of Medicine	1979 - 1989	131
Allen, 1999	Retrospective	United States	Rush-Presbyterian-St. Lukes Medical Center	1986-1994	117
O'Brien, 2005	Retrospective	United States	University of Pennsylvania School of Medicine	1992-2002	71

Langdon, 2016	Retrospective	United States	Boca Raton Regional Hospital	2002-2015	179
Balla, 2020	Retrospective	United States	Baylor College of Medicine	2001-2018	46
McDonald, 2003	Retrospective	United States	Washington University School of Medicine	1995-1999	246
Horr, 2017	Retrospective	United States	Cleveland Clinic	2000-2012	1281
Daugirdas, 1986	Prospective	United States	Loyola Medical center	Specific dates not mentioned	16
Dosios, 2003	Retrospective	Greece	Athens University Medical School	1991 - 2001	104
Porte, 1999	Retrospective	France	Calmette Hospital lille University Hospital	1985 - 1998	114
Wall, 1992	Prospective	United States	Duke University Medical Center	1987 - 1988	57
Georghiou, 2005	Prospective	Israel	Rabin Medical Center	2001 - 2004	18
Muhammad, 2011	Prospective	Saudi Arabia	King Fahad Hospital	2007 - 2009	30

Table S2. Newcastle ottawa scale for observational studies:

Studies	Selection	Comparability	Outcome
Cardak, 2023	***	*	**
Balla, 2020	***	**	***
Altman, 2015	***	**	***
Brien, 2005	***	**	***
Olson, 1995	***	**	***
Park, 1991	***	**	***
Salim, 2018	***	**	***
Horr, 2017	***	**	***
Langdon, 2016	***	**	***
Becit, 2003	***	**	***
Celik, 2012	***	**	***
McDonald, 2003	***	**	***
Sarigul, 1999	***	**	***
Allen, 1999	***	*	**
Moores, 1995	***	**	***
Wikes, 1995	***	**	***
Piehler, 1985	***	**	**
Palatianos, 1989	***	**	***
Olsen, 1991	***	**	**
Trigt, 1993	***	*	***
Naunheim, 1991	***	*	**
Degirmencioglu, 2022	***	**	**
Porte, 1999	***	**	***
Wall, 1992	***	**	***
Daugirdas, 1986	***	*	***
Georghiou, 2005	***	**	***
Dosios, 2003	***	**	***
Muhammad, 2011	***	**	***
